# Supporting Bees in Cities: How Bees Are Influenced by Local and Landscape Features

**DOI:** 10.3390/insects12020128

**Published:** 2021-02-02

**Authors:** Anthony C. Ayers, Sandra M. Rehan

**Affiliations:** Department of Biology, York University, Toronto, ON M3J 1P3, Canada; beeb0i@yorku.ca

**Keywords:** urban bees, functional traits, urban heat island effect, pollinator health, phylogenetic diversity, habitat fragmentation, microclimate, dietary breadth, green spaces, body size

## Abstract

**Simple Summary:**

Cities are complex ecosystems that, while generally contributing to an overall reduction in biodiversity, can support surprisingly unique communities of organisms including bees. Bees are both ecologically and economically essential, therefore preserving and conserving these insects represents a significant challenge as cities continue to expand and diminish surrounding landscapes. Some attempts to support bees in cities have included establishing and improving urban green spaces. Exactly how bees and, to a lesser extent, other pollinators respond to these green spaces in addition to other urban landscape and local features, however, remains incompletely understood. Therefore, this review summarizes the current literature and generalizable trends in pollinator response to urban landscape and local features. While some functional traits or characteristics of bees such as dietary breadth and nesting strategy are more conclusively understood and supported, other characteristics such as sociality remain less generalizable. Lack of knowledge on bee responses to city features is in part due to the individual variation exhibited across different groups and species. To promote greater biodiversity in urban spaces, research should focus on specific responses to urban local and landscape features and how green spaces can be optimized for sustainable bee conservation.

**Abstract:**

Urbanization is a major anthropogenic driver of decline for ecologically and economically important taxa including bees. Despite their generally negative impact on pollinators, cities can display a surprising degree of biodiversity compared to other landscapes. The pollinating communities found within these environments, however, tend to be filtered by interacting local and landscape features that comprise the urban matrix. Landscape and local features exert variable influence on pollinators within and across taxa, which ultimately affects community composition in such a way that contributes to functional trait homogenization and reduced phylogenetic diversity. Although previous results are not easily generalizable, bees and pollinators displaying functional trait characteristics such as polylectic diet, cavity-nesting behavior, and later emergence appear most abundant across different examined cities. To preserve particularly vulnerable species, most notably specialists that have become underrepresented within city communities, green spaces like parks and urban gardens have been examined as potential refuges. Such spaces are scattered across the urban matrix and vary in pollinator resource availability. Therefore, ensuring such spaces are optimized for pollinators is imperative. This review examines how urban features affect pollinators in addition to ways these green spaces can be manipulated to promote greater pollinator abundance and diversity.

## 1. Introduction

**Figure 1:**
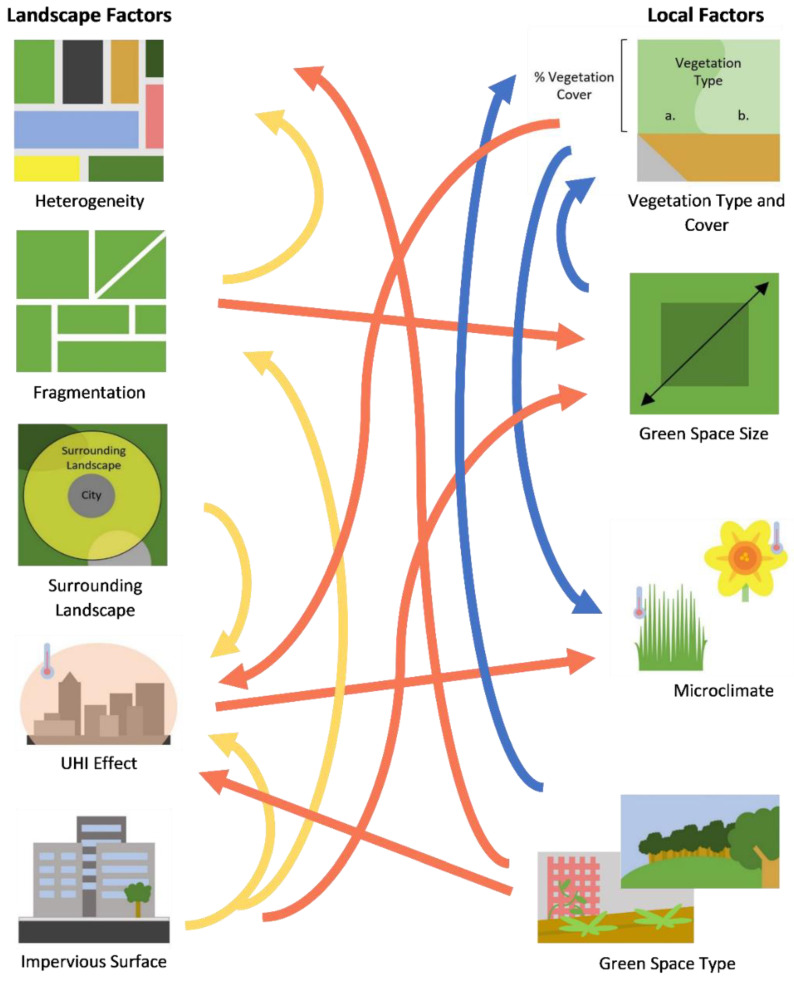
Diagram displaying some of the interactions between landscape and local features. Arrows represent features affected by other features from which the arrow originates. Local–local (blue), landscape–local (red), and landscape–landscape (yellow) interactions are shown.

**Figure 2:**
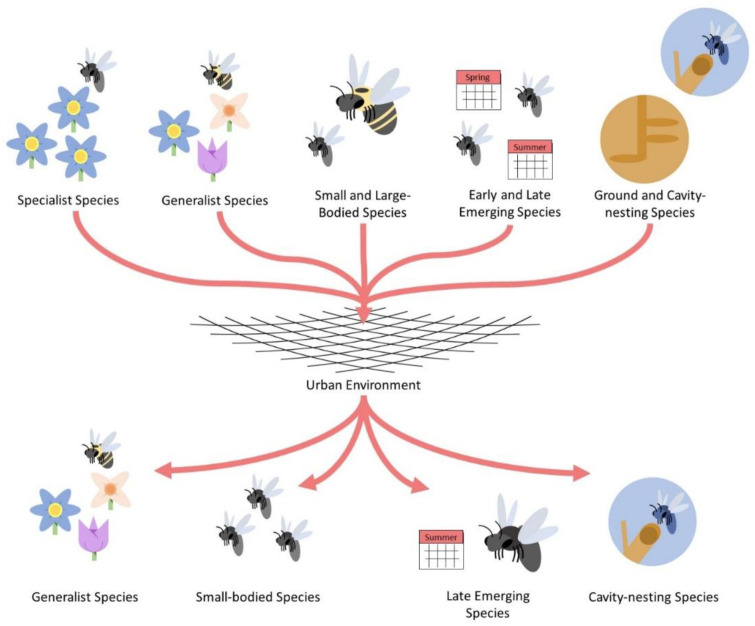
Simplistic diagram depicting the filtering of some functional traits comprising urban bee communities. The “net” in the center of the diagram represents a generic urban environment with all its associated local and landscape features that may influence bee community structure. General bee functional traits are presented at the top of the figure whereas traits selectively favored by cities are presented at the bottom.

Urbanization is a pernicious, anthropogenic alteration of the environment characterized by an increased proportion of impervious and built surfaces (roads, sidewalks, parking lots, buildings etc.), in addition to high human population density and total population size [1]. The development of urbanized areas, in conjunction with intensified agriculture, have been considered primary drivers of biodiversity loss, with declines noted for multiple insect taxa including coleopterans, lepidopterans, dipterans, and hymenopterans [2,3,4,5,6]. Reduction in biodiversity is often due to varying interactive elements composing the urban environment. These factors range from habitat loss and fragmentation, the presence of exotic plant and animal species, urban warming or the urban heat island (UHI) effect, and the reduction in habitat quality (Figure 1) [7]. These constituents of urbanized ecosystems can influence insect communities at either the landscape or local scale [8,9,10,11,12]. As a result, cities have been described as “filters” that influence the composition of these urban-dwelling communities by favoring particular functional traits, or organism characteristics that influence fitness, and life history strategies [13,14,15]. Consequently, urban filtering may homogenize communities according to functional traits characteristics, some of which are described in Figure 2 [16,17].

Despite the generally negative impact the urban matrix exerts on city species, urban areas can represent biodiverse ecosystems that serve as refuge for many ecologically important groups such as pollinators [10,18,19,20]. In fact, in some instances, cities may harbor greater diversity and abundance than surrounding agricultural and even semi-natural landscapes [21,22,23,24].

The diversity and abundance observed within cities are in part due to the heterogeneity of the urban landscape in which various habitat or green space types exist [6,25]. Urban green spaces like parks, gardens, and residential yards are notable habitat types that can provide essential foraging and nesting resources for bees and other taxa [26,27]. Urban green spaces have also been used to enhance habitat connectivity by establishing green space corridors or “stepping-stone” habitats [28]. The use of green spaces to promote biodiversity and mitigate urbanization impacts has been an area of increasing interest in recent years. Understanding how these and other urban spaces can be optimized to better promote and maintain species richness and abundance is essential, especially when acknowledging the future growth of urbanized areas needed to accommodate an increasing global population. According to the United Nations, approximately 55% of the 2018 world population lived in urban areas—a percentage that is projected to increase to 68% by the year 2050 as the population continues to expand to a staggering 9.8 billion people [29]. Canada and the US for instance exhibit high rates of urbanization, where >80% of the population can be found living in urban areas [30,31]. Insects are an important study group due to their rapid response to environmental change [32]. Specifically, ascertaining insect pollinator response to the individual components of urbanization is particularly crucial not only because declines have already been reported for these animals [33], but also because they provide essential ecosystem services: the most notable of which being pollination. Over 90% of flowering plants depend on animal-mediated pollination to some extent to successfully reproduce, a significant portion of which is facilitated by bees [34,35]. These services not only impact plant fecundity, but they also affect the organisms that rely on the fruits, seeds, and other resources resulting from such services. Humans are no exception to this, especially considering the scale of agriculture and food production today. As regions become more urbanized, agricultural practices have even begun to be incorporated into urban contexts, which also greatly depends on pollinators [36,37]. Visitations by wild pollinators, for instance, has been shown to increase the fruit and seed set in agricultural and urban contexts even more so than managed honey bees [38,39]. Therefore, wild pollinators can play an integral role in the economics of urban agriculture, in addition to potentially stabilizing food security in urban contexts.

The general appreciation of charismatic insects such as butterflies and bees amongst the public can also potentially be used as a means of facilitating community participation in and the promotion of insect conservation. For instance, a study by Southon et al. [40] examined community response to urban meadows and found that not only did participants show a preference for meadows over managed, mown areas, but tolerance for urban meadows increased when participants were informed of the benefits such habitats can provide for pollinators. A study by Pawelek et al. [41] also demonstrated a community’s willingness to participate in pollinator conservation as most gardeners displayed a desire to learn more about the pollinators visiting their community garden in addition to allowing the planting of flowers that can attract pollinators into their plots.

The purpose of this review is threefold: (1) To summarize the effectiveness of green spaces and other management and policy strategies that have been implemented in ameliorating pollinator losses. (2) To detail the facets of urbanization that affect pollinating insect traits such as species richness and abundance, body size, generality, sociality, nesting habitat, and behavior with a primary emphasis applied to bees since the bulk of the current literature has largely focused on bees. (3) To discuss gaps in knowledge that exist to inform future research so that a holistic understanding of urbanization impacts on pollinators can be established with the intent to generate more effective policy and management measures.

## 2. Landscape and Local Feature Influences on Pollinators

Different urban features variably influence pollinating insects, the effects of which are dependent upon the local and landscape features that constitute those specific environments. Landscape features pertain to the environmental characteristics that surround habitat spaces, whereas local features refer to the features of such spaces [12,42,43]. Some of the features in this article are not exclusive to either feature. For instance, impervious surface can be classified as either landscape or local features depending on the goals of a particular study [8,44]. Factors such as impervious surface may perhaps be more impactful from a landscape perspective and therefore will be treated as such throughout the course of this review. Of the two, local features are presumed to influence insect diversity the most [45,46]. Regardless, the improvement of surrounding landscapes in conjunction with local features can better enhance pollinator diversity [47,48]. The constituents relating to both landscape and local features will be discussed in further detail below in which knowledge gaps within each area will be addressed.

## 3. Landscape Features

### 3.1. Habitat Loss, Fragmentation and Heterogeneity

Fragmentation and habitat loss have been proposed as two of the most significant drivers contributing to bee species richness and abundance decline [49]. The development of buildings, roads, and other impervious surfaces across cityscapes produces a fragmented urban matrix containing habitats of reduced size and quality [50]. Both habitat loss and fragmentation also influence landscape composition and configuration in such a way that alters plant and pollinator densities in addition to pollinator movement and behavior [51]. The fragmentation of landscapes can complicate patch accessibility, especially for smaller bees with reduced mobility [7,52,53]. However, due to the probable different management practices implemented within each fragmented area, cities can become quite heterogeneous. Thus, the heterogeneity of the city landscape can produce idiosyncratic habitats featuring different resources.

### 3.2. Urban Heat Island Effect

Due to the pervasiveness of impervious surfaces throughout urban landscapes, cities typically exhibit warmer and drier local climates than surrounding locations [54]. This phenomenon, referred to as the urban heat island (UHI) effect, can elevate city temperatures 2–4 °C higher than rural areas, although one study has reported increases as high as 12 °C [55]. This substantial influence on city climate can affect plant community densities and advance their phenology in addition to affecting insect characteristics such as physiology and abundance. The UHI effect has facilitated the movement of other insects including the exotic wasp species *Sceliphron curvatum* into warmer urban regions [56]. Such movements of alien species may also occur among bees; however, further studies should be conducted as some reports have suggested urban warming has no influence on exotic bees [57]. This may make sense as many exotic bees found within cities are cavity-nesting and therefore nesting availability may be exerting greater influence on their presence [58].

The resultant changes associated with UHI effect affect different taxa variably [59]. Differential effects can occur interspecifically within the same taxa as demonstrated in butterflies displaying varying desiccation and thermal tolerances [60]. Recent research has used thermal maximum (CTmax) and critical water content (CWC) values as proxies for determining bee tolerance to urban warming [61,62]. The use of such metrics has revealed morpho-group specific responses to urban conditions including increased bumble bee thermal tolerance yet reduced desiccation tolerance compared to groups like sweat bees [60]. Ultimately, cities may be selectively favorable to bees and other pollinators possessing higher thermal tolerances than thermally susceptible species [60,61,62]. Depending on city location, the UHI effect may facilitate insect growth and development in regions that are regionally cooler, as postulated by Burdine et al. [61]. However, in warmer locations or in the future as climate change raises global temperatures, cities may push species closer to their thermal tolerances and ultimately affect their abundance [60]. For instance, studies in the US found that bee abundance decreased by 41% per 1 °C increase [62]. In this sense, the establishment of green spaces may provide an additional role in cities creating urban cooling island (UCI) effects in pocketed regions of cities [63,64]. Buildings themselves can produce UCI effects during certain times of day i.e., mornings; however, green spaces can absorb additional heat during warmer times of the day and year depending on how impervious and vegetation dense such spaces are [64,65].

### 3.3. Surrounding Landscape and Impervious Surface

The type of landscape surrounding an area can impact the presence of pollinators in a region, especially within agroecosystems where pollinator presence can influence fruit set [8,43,66,67]. In cities, it has also been predicted that higher species richness and abundance should occur at locations within closer proximity to natural areas and those furthest from urban cores [32]. Studies conducted in Poland and France have substantiated this hypothesis while examining urbanization impacts on wild bees along urbanization gradients [11,18]. Additionally, increasing the proportion of green spaces in the surrounding landscape can increase the richness and abundance of bees [68]. Furthermore, the presence of nearby semi-natural areas and green spaces within urban areas can be especially useful for pollinator visitations as these resources enhance the total number of resources that can be utilized within or near landscapes [69]. Semi-natural areas surrounding orchards, for instance, possess gastropod shells that can be repurposed by bees to serve as nests [70]. It is possible that maintaining the semi-natural areas surrounding cities may also preserve resources necessary for some bees that are found near urban areas to persist.

Urban areas tend to be surrounded by and diffused with impervious surfaces. Several studies have investigated impervious influence on pollinator abundance, species richness, and community structure using urbanization gradients [4,5,10,11,71]. Despite recent criticisms regarding the use of imperviousness as a defining metric for urbanization, its use has become relatively standardized within urban pollinator ecology [15]. Inconsistencies in the classification methods may affect results in such a way that over or underestimates the actual urban impact on pollinators. Regardless, species richness and abundance generally appear to decrease in association with increased proportions of impervious surfaces [6,72]. Intensification of impervious surfaces has also been associated with shifts in pollinator community structure where greater proportions of impervious surface generally possess a higher abundance of cavity-nesting bees compared to ground-nesting bees [11,72,73]. Such results appear intuitive, given that impervious surfaces diminish the amount of available nesting space that can be utilized by ground-nesting insects; however, such trends are not entirely generalizable as city compositions may vary uniquely to affect bees differently. Studies should be conducted to further substantiate the filtering out of pollinators that nest in the ground.

## 4. Local Features

### 4.1. Microclimate

Microclimates, or local climatic conditions within habitats, can vary across urban environments and even within individual habitat patches depending on the biotic and abiotic qualities of a space [13,74,75]. Vegetation type and cover along with background environmental warming are two such characteristics that can influence local microclimate [76]. Vegetation can generally reduce surrounding temperature [64] and at an even finer scale, flowers including daffodils possess independent microclimates which, in some cases, act as warming pads for bees [77]. Shade produced by vegetation cover or other environmental factors can also influence the microclimates of nests used by cavity-nesting bees [78]. Such microclimate effects within the nest can influence the metabolic rate and development of bee larvae [78,79]. Abiotic factors i.e., nitrogen deposition, which is prevalent in urban areas, can interact synergistically with climate change in such a way that may influence local microclimates as well [7,80]. Both factors can hasten spring plant growth (a factor that reduces microclimate temperatures) which has been previously shown to ultimately affect the developmental success of thermophilic butterflies by reducing their abundance [80]. Such a pattern may also be observed in bees and other pollinators. Despite the knowledge that landscapes can display heterogeneous microclimates, few studies have conducted microclimate studies at extremely fine scales within cities [61,62]. Understanding how species respond to specific habitat variables is essential as shifts in microclimate may impact pollinator populations either through extirpation or dispersal.

### 4.2. Urban Vegetation

Exotic and ornamental plant species can be found extensively throughout urban areas especially within residential yards and gardens [81]. This increase in plant variety contributes to the increased species richness and/or abundance of plant communities which may enhance the species richness of pollinators and other insects [26,28,53,58,82]. This correlation obviously largely depends on increasing the species of plants attractive to bees and lepidopterans rather than just generally increasing plant richness [83]. Floral abundance and frequency, however, may be more impactful than promoting richness itself [84,85]. Other factors including increased floral area (area occupied by blooming plants) have also been shown to influence butterfly and bee abundance [45,86].

Due to the high prevalence of exotic plants within urban areas, determining non-native influence on the local environment and native pollinators is important. Previous reviews have suggested that exotic plants can negatively affect pollinator communities by influencing visitations and the reproductive success of native plant species [87]. A more recent review, however, indicated that non-native plants are not preferred over native plants among pollinators [7]. This lack of preference was, in part, attributed to potential design flaws in the approach of several previous studies [7]. Although preferences may not vary drastically among generalists, oligolectic species associated with few or a single native species are particularly vulnerable to alterations in plant communities [88]. Therefore, preserving native plant species is one essential aspect of sustaining specialist, native bees. Additional research suggests, however, that exotic species may negligibly affect pollinator presence [89,90,91]. In fact, exotic plants may benefit areas by extending floral resource availability throughout the season and contributing to the overall abundance of floral resources [92]. Due to these seemingly conflicting results, further attention should be directed towards understanding the influence exotic plants possess over pollinators more conclusively. This is especially apparent given that some evidence suggests that native and perennial plants exhibit greater bee abundance compared to exotic and annual species [42,93].

### 4.3. Green Space Size

Habitat size is an important factor potentially capable of influencing pollinator presence by limiting the quantity of resources able to occupy green spaces. In order to sufficiently accommodate adequate amounts of floral and nesting resources necessary for pollinators, green spaces must be of adequate size. However, the total area needed to support pollinators remains poorly understood and is perhaps dependent upon bee mobility [52,94,95]. Current research conducted on habitat area primarily investigates components of green space area like the proportion of floral cover and, to an extremely lesser extent, nesting resource availability [70]. Beninde et al. [9] examined space requirements for bees and indicated that areas larger than 50 ha should be established to prevent losses in species richness. Too much green area may not provide any particular benefit for certain taxa though as bee diversity has in some instances plateaued outside of a 100-m buffer radius [53]. Regardless, establishing larger habitat areas has been shown to positively affect butterfly and bee species richness, abundance, and diversity [85,96,97,98]. Such variations in size may be more influential on the richness and abundance of smaller bees than large bees, such as *Bombus* spp., which exhibit greater flight distances and increased mobility associated with size [99]. Nevertheless, creating spaces of both sustainable size and resource availability are important considerations when attempting to mitigate habitat fragmentation and increase connectivity, especially for less mobile, smaller species [100].

### 4.4. Green Space/Habitat Type

Habitat types can differ in their ability to support pollinating insects due to variation in site-specific management practices and resource availability. For instance, green spaces like remnant vegetation, urban parks, residential neighborhoods, and golf courses exhibit variability in plant species composition and vegetation cover which can influence their resourcefulness to pollinators [24,27,68,81]. The availability of resources at specific green spaces depends on what the functions of those sites are. Residential yards tend to be maintained for aesthetic value and, therefore, implement vigorous management regimes such as frequent mowing that negatively influence bee abundance and diversity through the removal of weeds and other useful flower resources [101]. Green spaces like urban grasslands, which may experience less frequent management, have been found to contain higher butterfly, hoverfly, and bee abundance compared to urban parks and housing estates which is perhaps unsurprising as reduced management preserves pollinator floral and nesting [102]. Other random wildflower patches can also be particularly important as they can host ground nesting bees such as *Andrenidae* spp. despite occasional mowing [24]. Urban gardens can also be hotspots for bee abundance and functional trait diversity perhaps due to the presence of a variety of plant species, many of which are reliant upon pollinators [25,26,45,84,103]. Normandin et al. [103] found that, in some instances, community gardens are only slightly less efficient in attracting bees compared to urban parks despite being typically smaller spaces.

Different park types can also vary in their ability to support pollinators. Recreational parks for example could improve floral quality [104] and planting gardens within these areas may be one means of accomplishing such a task [105]. Informal green spaces including those found interspersed throughout residential areas may be useful to pollinators if they exhibit variation in management intensity [106]. Other urban areas including brownfields, which represent previously developed urban industrial locations, may also be repurposed in ways that promote bee biodiversity [107] especially if sites are not entirely isolated and possess available foraging resources [108,109]. Due to lack of use, derelict and other post-industrial sites may also be of interest as they can be unmanaged and experience reduced pesticide and/or chemical exposure, unless located at former chemical plants [108,110]. Currently, few studies have concurrently examined the quality of multiple green spaces, often focusing on one or a few specific types [103,111]. Examining the value of multiple green spaces within an urban environment is important to understand which spaces are especially beneficial for their conservation value and which should be investigated further to improve their overall habitat quality.

## 5. Functional Traits Affected by Urbanization

Urbanization has been previously described as a filter that selectively favors pollinators possessing functional traits which render such taxa less vulnerable to heavily disturbed environments [13,14,15]. Despite a city’s ability to support a breadth of biodiversity, the filtering associated with cities may produce homogenized communities based upon functional traits or phylogenetic relatedness (the summary of which can be found in Table 1) [7,17,21,83]. Various studies have noted several similar trends regarding urban bee taxa and functional traits, however particular observations may not be easily generalizable due to species specific variation in response [111,112]. Amongst pollinators, general functional traits of taxa observed in urban areas pertain to differences in dietary breadth, nesting strategy, body size, behavior via phenology or sociality, and phylogenetic diversity.

### 5.1. Dietary Breadth

Most studies that have examined species composition across urban areas have reported consistent, relatively general trends regarding pollinator lecty or dietary breadth. There is ample evidence to suggest that cities support greater abundances of polylectic (i.e., generalist) pollinators displaying broader foraging preferences compared to oligolectic (i.e., specialist) species displaying limited dietary preferences restricted to one or few plant hosts [11,18,71,84,103]. Although specialists are under-represented and even rare in cities [113], their presence is not entirely lacking, particularly if species-specific requirements are available within the landscape [84,114].

Urban environments may inadvertently advantage generalist species as floral landscapes, although diverse, are largely dominated by exotic, ornamental and other species which are not hosts to specialists [81,115,116]. Flowers possessing deeper corollas may limit resource availability for bees and other pollinators possessing short tongues by being inaccessible to such species [117]. The lack of host plant species in an area may thus effectively turn urban spaces into resource deserts for specialists. Understanding the specific associations between pollinators and the plants they utilize is important if appropriate and effective conservation measures are to effectively preserve target species such as specialists. Currently, some studies examining pollinator trends still fail to record species-specific interactions at the expense of using morpho-group classifications in the field [11,21]. While the use of morpho-groups provides relevant information about plant–pollinator relationships, interactions identified to the species will be most informative of the diversity and specificity of relationships existing between plants and pollinators.

### 5.2. Nesting Strategy

Nesting strategy primarily applies to bees as the nesting of other pollinators such as butterflies and syrphids is dependent on the surrounding vegetation. Bees are broadly categorized as either subterranean ground-nesters or above-ground cavity-nesters. The vast majority of the >20,400 bee species distributed world-wide are ground-nesting; however, cavity-nesting bees appear overrepresented across many urban studies likely as a result of the conversion of bare ground into impervious surface [11,73,83,111,118]. Additionally, urban spaces tend to possess novel, above-ground nesting resources such as cracks and holes in structures and, to a lesser extent, bee hotels that can be exploited by above-ground nesters [119]. For example, *Xylocopa virginica* is a species found in proximity of anthropogenic disturbance having now adapted to using milled lumber as a nesting substrate [120]. Community gardens and urban park sites may promote ground-nesting species, however, as these green spaces often contain bare soil and stem-nesting substrate [84].

### 5.3. Body Size

The effects of urbanization on pollinator body size has received conflicting results within the literature despite some suggestion that cities may promote smaller body sizes [18]. While some studies observe greater proportions of large-bodied pollinators including both bees and butterflies, others notice an opposite or undetectable trend across urban landscapes [121,122]. Since flight distance for pollinators such as bees has been correlated with body size, it has been assumed that large-bodied bees, for instance *Bombus*, are able to navigate between patches more efficaciously than smaller-bodied bees including *Lasioglossum*, which exhibit shorter flight ranges [52,99]. In semi-natural grasslands, it is suspected that this greater mobility associated with size explains the higher species richness of larger bees [98]. This trend may be paralleled in cities where a greater abundance of larger pollinators such as butterflies has also been reported [72].

Despite the greater mobility of larger species, however, smaller species may require less resources to sustain themselves in urban areas [73,121]. Additionally, factors such as UHI effects and reduced nutrition availability could be driving reduced body size in traditionally larger pollinators such as *Bombus lapidaries* and *B. pascuorum* [123]; however, other sources have reported the opposite effect in *B. terrestris* better attributed to fragmentation. It could be the case that different factors not only idiosyncratically influence body size but do so in a way that varies intra and inter-specifically across varying taxa as well [124,125]. Additional studies are needed to better disentangle which urban features are more greatly impacting body size.

### 5.4. Behavior

Urbanization affects several pollinator behavioral traits ranging from foraging and nesting decisions, anti-predator response, to potentially filtering species based on their degree of sociality. Through factors such as the microclimate, potential nest sites in cities may alter the nest-site decisions of some bees as was observed in *Megachile rotundata.* In this study using artificial nest boxes, tubes possessing lower temperatures were occupied more frequently than those displaying higher temperatures [126]. Several studies evaluating foraging decisions in urban environments or across urbanization gradients typically do so using plant–pollinator interaction networks. The degree of urbanization can influence these interaction networks and visitation rates between wild bees and available urban plants. Sweep netting across urban locations has revealed preference for native floral resources and reduced visitation to exotic plants [127].

Whether solitary or social species are most prevalent across urban landscapes remains inconclusive within the literature. While some studies report a greater abundance of solitary bee species [83], others indicate that social species appear most frequently in urban areas [15,22,128]. A systemic review analyzing urban bee functional traits, however, found no generalizable trend for sociality [111]. Discretions in results could be a result of contrasting methodologies. For example, Wilson and Jamieson [58] incorporated cleptoparasitic and sub-social bees into their solitary category whereas other studies may examine these group separately. Future studies should continue to examine urban effects on functional traits including sociality, but as found with most other traits, trends may be regionally specific and not generalizable due to the variation of response occurring across bee species and the variation of study regions themselves [112].

When dissecting the urbanization impact on pollinator phenology, some work suggests that cities may support later season bees more so than early emerging species [15,18]. Such changes are likely a result of climatic changes which can produce mismatches between flowering plants and pollinators [129]. Bees that display greater adaptive response to phenological shifts, like some social bees with prolonged foraging seasons, may be able to take advantage of resources more effectively than species with shorter active seasons [7].

### 5.5. Phylogenetic Diversity

Although many studies tend to focus on functional trait homogenization, recent work has investigated similarities from an evolutionary perspective [130,131]. Phylogenetic diversity may be correlated with functional traits; phylogenetic diversity changes may be more useful than functional trait changes in more accurately reflecting taxonomic changes within a community [130,132]. Phylogenetic studies in conjunction with functional trait studies may also be additionally useful in further determining vulnerable urban species within urban environments [133]. Despite the potential benefit associated with phylogenetically based studies, few have occurred within urban contexts. Such approaches implemented in cities, however, were able to detect family level phylogenetic homogenization, or predominance of few families over others, even when species homogenization was not apparent [130]. The results of Harrison et al. (2018) indicate that urban areas within the New Jersey region possess phylogenetic homogenization represented by communities dominated by bees within the Halictidae family. Ascertaining shifts in phylogenetic diversity resulting from urban land use is imperative, especially considering the reduction in such diversity equates to a loss of bee evolutionary history and a reduction in ecosystem services [134]. Former phylogenetic studies have noted that genera such as *Lasioglossum (Dialictus)* and *Bombus* are more abundant/resilient than others, such as *Andrena* and *Nomada* who may be particularly sensitive to land-use change [103,130,131,134,135]. Such clades may be increasingly vulnerable to urbanization due to their specialized diets, earlier and/or shorter flight seasons, and other characteristics that are phylogenetically correlated [130]. Additionally, some vulnerable bee guilds, such as cleptoparasites, may be useful in serving as bio-indicators of community health [136].

**Table 1:** Summarization of some of the functional traits affected by some of the varying urban factors presented throughout the review.

Functional Trait	Favored Strategy	Urban Factors Affecting Traits	References
Body size	Inconclusive (mixed results)	UHI effect, Habitat fragmentation, Vegetation cover and type	[71,121,123,125]
Diet strategy	Polylecty	Green space type, Vegetation type and cover	[11,18,70,84,103]
Nesting strategy	Cavity nesting	Impervious service, Green space type, Green space size	[26,70,71,77]
Phenology	Late emerging	UHI effect, Vegetation type and cover	[15,18,129]
Sociality	Inconclusive (mixed results)	Impervious surface, Vegetation cover and type	[15,18,22,111,128]
Phylogenetic Diversity	Groups such as *Lasioglossum (Dialictus)*, in some instances *Bombus*	Impervious surface, Vegetation cover and type, UHI effect, Fragmentation, Green space size	[103,130,131,134,135]

## 6. Conservation Aims and Future Directions

Conservation initiatives within urban areas aimed at minimizing pollinator declines largely include the establishment and protection of green spaces throughout the urban matrix. Ensuring that these dedicated locations, which may differ in resources depending on the type of space, sufficiently provide quality resources directed towards multiple pollinating taxa is essential considering the different needs of pollinators. While some studies provide recommendations on how to modify spaces for pollinators, striking a balance between appeasing both the public and stakeholders while achieving conservation goals remains a complex issue [94,137]. The economic and political component of conservation and restoration, however, may be especially challenging as policymakers may be reluctant to implement costly changes that do not administer immediate results [138].

The public often holds considerable influence in selecting plant species, which are decisions made based on aesthetic value [139,140]. Additionally, green space approval and preferences amongst the public depends on socioeconomic conditions such as median house income, gender, and level of education for example [26,40,141]. The inclusion and/or preservation of green areas may not solely benefit pollinators; however, as positive human health benefits have been associated with green space presence [142,143,144]. Green space exposure has previously been suggested to be negatively associated with health factors such as mortality and positively associated with factors including physical activity and some aspects of mental health [145]. Additionally, green spaces such as urban gardens can reconnect individuals with nature and encourage community engagement of conservation objectives [41,84,146].

Economic costs typically hold precedence over ecological and recreational concerns [147]. As a result, effective measures should be developed to ensure that environmental projects are not only of high-quality and meet expectations but are also cost-effective. Despite any hesitancy expressed by potential stakeholders, the inclusion of green spaces and/or modified management practices may reduce total costs over time. For instance, a reduction in intensive management practices such as excessive and frequent mowing not only benefits pollinators by preserving floral resources but also reduces costs associated with mowing [82,101,148] while maintaining site aesthetic value [40]. Additionally, the inclusion of perennial plants may also reduce management costs by mitigating the frequency at which seeds need to be re-sown in comparison with more labor-intensive annuals [149].

Improving upon plant and seed mix selections placed within green spaces could also enhance habitat quality and prevent ineffective spending. Seed mixes applied in urban settings can undoubtedly attract pollinators such as bumble bees and hoverflies; however, the plant species incorporated into such mixes are important. The inclusion of sometimes specific plant species can greatly enhance pollinator visitation rates within habitats [41]. Several plant species used in current seed mixes, sold in stores, and generally found in urban spaces may be of little value to pollinators, and those that are described as pollinator-attracting plant species lack empirical evidence [91,115,150]. Since pollinators exhibit a wide variety of dietary preferences, elucidating species-scale preferences is imperative to maintain greater aspects of pollinator communities [137].

Establishing plant–pollinator interaction networks could be one method of empirically determining species resolution pollinator resource usage and floral visitation as done in bumble bees [151]. These networks are typically constructed using field-recorded observations or pollen extractions and direct bee sampling from flowers [93,117,152]. Additionally, such networks have previously illustrated that the inclusion and/or exclusion of plants incorporated in seed mixes used in prairie restoration can significantly affect bee richness and abundance [150]. Constructed networks can then depict how different pollinators depend on different floral resources and show how network characteristics such as generality can shift across urban landscapes. Not only can this be important for determining if certain pollinating groups, especially oligolectic specialists, have sufficient floral resources, but conversely, interaction networks can also indicate whether a plant species is receiving sufficient pollinating visitors.

The successful implementation of management and other pollinator protection strategies, however, in part greatly depends on the passage of appropriate legislature from national and regional governments. While pollinator-protecting legislature has only until recently begun to gain traction, many countries have made several steps to improve efforts. For instance, 110 laws associated with pollinators have been passed at the US state-level from 2000 to 2017 [153]. Actions within cities have begun taking place, as the City of Toronto government, for example, has developed a pollinator protection strategy to promote pollinator awareness and conservation [154]. Invertebrate conservation groups such as the Xerces Society have recognized cities within the United States and Canada that promote pollinator biodiversity as “Bee Cities” [155]. Not only can the passage of such policies provide some direct degree of ensured protection for pollinators, but they can be used to garner public support and promote community engagement and learning [110].

In order to accurately assess the extent to which urbanization affects pollinators, researchers should modify their general approaches to urban research. To start, long term studies should be established over those lasting only a few years so that more stable results and trends can be ascertained. Additionally, studies should place greater emphasis on the South American, Asia, and Australian continents as the current literature is overrepresented by North American and European studies [4,13,15,111]. As a result, future studies should direct their attention to understudied regions of the world as results may not be easily extrapolated to such locations [85].

## 7. Conclusions

Although urban landscapes may more broadly reduce pollinator abundance [5] and favor particular functional and taxonomic traits over others [18], cities can still hold value as pollinator habitats [19]. Such shifts in pollinating communities, which support traits such as polylecty and cavity-nesting, are consequences of interacting local and landscape features which exert differential, non-generalizable effects on species within and across taxa [18,73,106,112]. Therefore, disentangling these urban effects on pollinators is important to understand how cities can best support pollinating communities.

Green spaces able to execute informed design principles and management schemes may effectively thwart pollinator biodiversity losses. However, implemented green spaces should be structured in such a way that meets pollinator and other vulnerable target group demands. Determining which aspects are most important to integrate requires further study. In the meantime, landowners should consider restorative measures including, but not limited to, reducing management intensity [100], promoting native floral abundance and richness [28], and establishing bare patch areas for ground-nesting bees [73]. Cities themselves should seek to green roofs and other green infrastructure to reduce fragmentation and promote connectedness, pollinator resource availability and pollinator movement [9,28].

Establishing a general scientific consensus on urban pollinator ecology is also imperative if implemented policy guidelines are to be successful in promoting pollinator biodiversity and appealing to both policy makers and the public. If a single party remains unmotivated to act, pollinator health may continue to decline. Science communication in this regard is especially important to ensure meaningful change can occur collaboratively. This approach should, of course, not solely apply to pollinators but to all threatened biodiversity. Pollinators, however, can serve as an important group to vocalize conservation initiatives amongst the public and foster the healthy co-existence of nature and urban society.

## Data Availability

No new data were created or analyzed in this study. Data sharing is not applicable to this article.
